# Limited change in adolescent value-based risk choices following acute sleep restriction and caffeine

**DOI:** 10.3389/fpsyg.2026.1879309

**Published:** 2026-08-12

**Authors:** Lina T. Lane, Noëmi C. Capdevila, Ann-Sophie Loock, Elias Mattern, Christian Cajochen, Carolin F. Reichert

**Affiliations:** 1 Centre for Chronobiology, Psychiatric Hospital of the University of Basel, Basel, Switzerland; 2 Research Cluster Molecular and Cognitive Neurosciences, University of Basel, Basel, Switzerland

**Keywords:** adolescence, Bayesian inference, caffeine, risk decision-making, risk-taking, sleep restriction, value-based decision-making, Wheel of Fortune

## Abstract

**Purpose:**

Adolescence is marked by changes in brain connectivity linked to risk decision-making, indexed by risk-taking propensity, optimal choices (safe, higher expected value), and irrational choices (risky, lower expected value). Insufficient sleep and caffeine consumption, common during adolescence, may independently and jointly influence risk decision-making in adolescents already predisposed to risk-taking. This study investigated whether sleep-restriction impairs risk decision-making, whether caffeine increases risk-taking propensity and optimal choices while decreasing irrational choices, and whether caffeine effects are enhanced under sleep-restriction.

**Methods:**

Forty-one adolescents aged 14–17 years completed a between-subject, two-night sleep manipulation (sleep-restriction: 6 h time in bed, sleep-extension: 9.5 h time in bed), followed by a within-subject, double-blind, placebo-controlled caffeine manipulation. Risk decision-making was assessed using a modified Wheel of Fortune task, and Bayesian inference quantified sleep and caffeine effects.

**Results:**

Across all outcomes, posterior estimates for sleep condition, caffeine, and their interaction were small, with 95% highest density intervals spanning zero; region of practical equivalence overlap varied across predictors, indicating limited evidence for practically meaningful effects. Caffeine effects and sleep-caffeine interactions remained uncertain across outcomes.

**Discussion:**

Adolescent risk decision-making on the modified Wheel of Fortune task showed no clear evidence of practically meaningful average changes after two nights of sleep-restriction or acute caffeine. These results suggest limited sensitivity of this task to the present manipulations rather than evidence that sleep or caffeine is inconsequential. Findings provide quantitative effect size and uncertainty benchmarks for future experimental work.

**Clinical trial registration:**

https://www.humanforschung-schweiz.ch/en/trial-search/study-detail/58864/translate/en/, identifier SNCTP000004988.

## Introduction

Teenagers demonstrate greater propensity for risk-taking than adults, a pattern linked to adverse health and developmental outcomes (Duell et al., 2018). This susceptibility is exacerbated by insufficient sleep, which is common in adolescents and partly driven by misalignment between biological rhythms and social demands (Short and Weber, 2018). This discord arises from the maturational delay of the sleep window (Carskadon, 2011; Jenni et al., 2005), a shallower build-up of sleep pressure (Jenni et al., 2005), early school start times (Nahmod et al., 2019), stable sleep needs of 8–10 h (Short et al., 2018), and increasing autonomy (Tashjian et al., 2019). Insufficient sleep has been associated with up to a 143% increase in maladaptive risk-taking behaviours (Short and Weber, 2018). Increases in delinquency, substance use, alcohol consumption, and risky sexual behaviour (Short and Weber, 2018) suggest disruption in risk evaluation and choice. To address the cognitive (de Bruin et al., 2017), emotional (Palmer and Alfano, 2017), and behavioural (Banks and Dinges, 2007) detriments of insufficient sleep, many teens consume caffeine (Mahoney et al., 2019), encouraged by marketing promoting enhancing effects on physical and cognitive performance (Heckman et al., 2010), despite limited evidence. This legal, affordable psychostimulant, used regularly by 80% of teenagers in high-income countries (Reichert et al., 2021), may increase risk-taking (Temple et al., 2017). Yet research has focused largely on adults (Bonnar and Gradisar, 2015), and given adolescents’ distinct physiological and psychological profiles, accurate data on caffeine’s effects and safety in this group are needed to inform policy and practice. This underscores the need to examine neurodevelopmental processes shaping adolescents’ vulnerability in risk decision-making (RDM). In this study, poorer RDM is indexed by greater risk-taking propensity, fewer optimal choices (safe, higher expected value), and higher irrational choices (risky, lower expected value).

During puberty, hormonal maturation (van Duijvenvoorde et al., 2016) and delayed development of prefrontal control (Ordaz et al., 2013; Van Leijenhorst et al., 2010) relative to subcortical reward valuation (Haber, 2011; Arain et al., 2013) heighten the influence of reward and affective processes on choice, an imbalance proposed to render adolescents’ value-based decision-making more vulnerable than adults’ to acute perturbations of sleep and arousal.

Insufficient sleep has been shown to impair prefrontal control processes in adolescents (de Bruin et al., 2017), which may increase vulnerability to poor RDM by further weakening top-down regulatory control over reward-driven behaviour and thereby increase risk-taking propensity, that is, a greater and more impulsive willingness to gamble regardless of value. Sleep loss may separately bias processing from reflective towards faster, more error-prone intuitive decision-making, impairing the integration of value information and thereby reducing optimal choice propensity (the selection of higher expected-value options) and increasing irrational choice propensity (value-insensitive gambling); this shift appears most pronounced under time pressure and may therefore depend on task demands (Fu et al., 2026). Epidemiological research consistently links insufficient sleep and increased real-world risk-taking behaviours in adolescents (Short and Weber, 2018). However, although sleep loss and RDM have been reviewed recently (Wei et al., 2025), experimental laboratory studies examining the association between sleep-restriction (SR) and RDM in adolescents are scarce. One of the few experimental within-subject studies in adolescents, conducted by Davis et al. (2013), investigated risk-taking during pedestrian street-crossing decisions in a virtual environment, comparing SR (4 h time in bed) with adequate sleep (8.5 h time in bed) in 55 adolescents (mean age 14.89, aged 14–15; 58% female). Sleep was manipulated in participants’ own homes with rest-activity cycles tracked by actigraphy; this captures everyday conditions but affords less control and greater between-participant variability. Four hours may not represent an ecologically plausible level of SR as many adolescents sleep 6–7 h on school nights (Singh et al., 2023; Wolfson and Carskadon, 1998), and the study’s 8.5 h ‘adequate sleep’ condition falls short of the up-to-10-h adolescent sleep need (Short et al., 2018). Results showed that sleep-restricted adolescents exhibited riskier behaviours (Davis et al., 2013).

Evidence on caffeine is also limited. Several correlational studies report associations between caffeine consumption and increased risk-taking propensity in adolescents (Kristjansson et al., 2018; Arria et al., 2014). However, only one study experimentally examines caffeine’s effects on adolescent risk-taking (Temple et al., 2017). Temple et al. (2017) found dose- and habit-dependent effects, with moderate users showing increased risk-taking at 2 mg/kg, high users reduced risk-taking, and low users showed no effect. Caffeine may thus modulate adolescent risk-taking, though mechanisms and boundary conditions remain unclear, and interpretation of these results is complicated by uncharacterised baseline differences between habitual-use groups, including age (Figner et al., 2009), sex (Byrnes et al., 1999), and pre-existing risk-taking tendencies (Caspi et al., 1997). More broadly, caffeine is known to influence arousal, attention, and cognitive control, processes central to value-based decision-making (Diukova et al., 2012). Through these effects on arousal and attention, caffeine may heighten sensitivity to reward and bias the evaluation of risky, higher-value options, which would be expected to increase risk-taking propensity, although the supporting human literature is limited (Ferré, 2016). Through enhanced cognitive control, by contrast, caffeine may support the integration of value information and improve decision quality, increasing optimal choices and reducing irrational choices. These predictions are not contradictory: because risk-taking propensity indexes willingness to gamble independently of value whereas optimal and irrational choices index value sensitivity, caffeine could increase gambling while sharpening value-based discrimination. These effects may be particularly relevant under SR, where caffeine could partially compensate for sleep-loss-related impairments in cognitive control and valuation, whereas under well-rested conditions its impact may be attenuated (Galli et al., 2022). Taken together, evidence suggests that caffeine alters risk-taking propensity and modulates decision quality in adolescents, but experimental findings are limited. More broadly, because the behavioural effects of sleep loss are task-dependent, small or null effects within a single paradigm are themselves informative, helping to delineate the boundary conditions under which SR does and does not alter value-based choice (Wei et al., 2025; Lim et al., 2025).

Based on the evidence reviewed, it was hypothesised that SR, compared with sleep-extension (SE), would be associated with poorer RDM, reflected in increased risk-taking propensity, reduced optimal choices, and increased irrational choices; that caffeine, compared with placebo, would increase risk-taking propensity while increasing optimal and reducing irrational choices; and that these caffeine effects would be more pronounced under SR than SE.

## Methods

### Study design

This study employed a mixed experimental design with a within-subject, double-blind, placebo-controlled caffeine manipulation and a between-subject manipulation of sleep condition to investigate effects on adolescent RDM measured using a modified Wheel of Fortune task (WOF) (Blankenstein et al., 2017; Blankenstein et al., 2018). This study was preregistered on the Open Science Framework.^1^

### Ethics

Ethics approval was granted by the Ethics Committee Northwest and Central Switzerland (BASECC2022-00930) and conformed to the Declaration of Helsinki. All participants provided written informed consent. The study was registered on the Swiss National Clinical Trials Portal, SNCTP000004988 and supported by the Swiss National Science Foundation (10001C_205164/1).

### Participants

**Figure 1:**
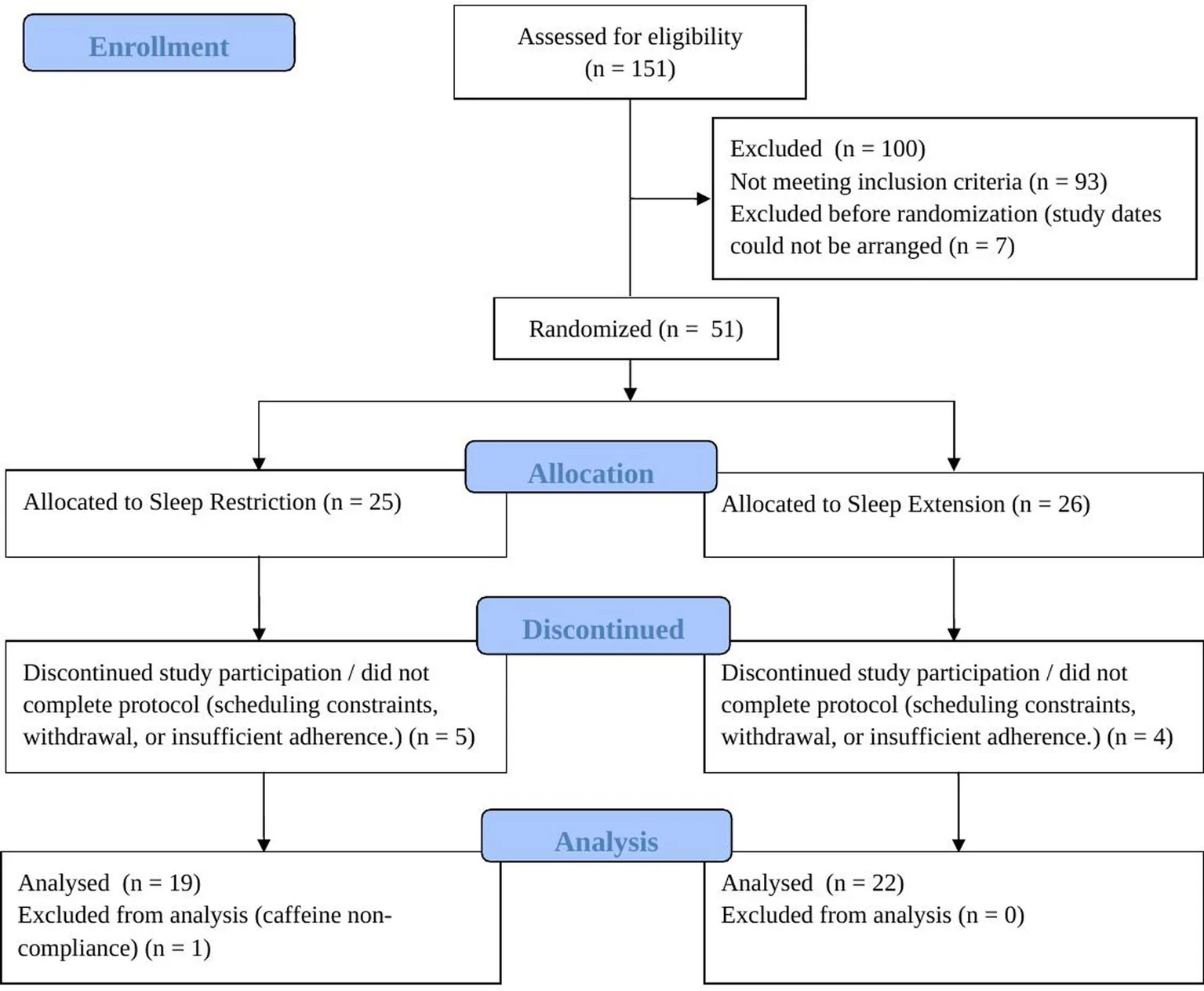
CONSORT 2010 flow diagram.

Participants aged 14–17 were recruited via convenience sampling (e.g., social media advertisements on Instagram) for a two-week clinical trial running from March 2023 to April 2025. Sample size was determined sequentially using Bayesian inference until evidence supported or refuted an effect (Vasishth et al., 2023), or until the April 2025 data collection deadline. Of 151 individuals screened, 58 were eligible; 7 were not randomised because dates could not be arranged. Fifty-one were randomised to SR or SE, of whom 9 did not complete the study (5 SR, 4 SE), reflecting scheduling constraints, withdrawal, or insufficient adherence. One further SR participant was excluded for caffeine non-compliance, giving a final analytic sample of 41 (19 SR, 22 SE). Participant flow is summarised in Table 1 and Figure 1 (CONSORT 2010 Flow Diagram) and characteristics by sleep condition in Table 2.

**Table 1:** CONSORT-style participant flow through eligibility, randomisation, study completion, and analysis.

CONSORT section	Flow item	Total *n*	SR *n*	SE *n*	Details/reason
Enrollment	Assessed for eligibility	151			Individuals screened
Enrollment	Excluded before eligibility/randomisation	93			Did not meet inclusion criteria, declined participation, or other screening-related reasons
Enrollment	Met inclusion criteria	58			Eligible individuals
Enrollment	Eligible but not randomised	7			Study dates could not be arranged or participation did not proceed before randomisation
Randomisation	Randomised to sleep condition	51	25	26	Randomised to SR or SE
Allocation	Allocated to SR, 6 h time in bed	25	25		Received assigned sleep condition unless later discontinued
Allocation	Allocated to SE, 9.5 h time in bed	26		26	Received assigned sleep condition unless later discontinued
Follow-up	Did not complete full study	9	5	4	Scheduling constraints, withdrawal, or insufficient adherence to protocol
Follow-up	Did not complete full study, SR	5	5		Old male: 1; young female: 1; old female: 3
Follow-up	Did not complete full study, SE	4		4	Young male: 3; old female: 1
Follow-up	Completed full study	42	20	22	Completed both study weeks
Analysis	Excluded from data analysis	1	1	0	SR participant excluded due to caffeine non-compliance
Analysis	Included in final analytic sample	41	19	22	Final analysed sample
Analysis	Analysed in SR condition	19	19		Included in final analysis
Analysis	Analysed in SE condition	22		22	Included in final analysis

**Table 2:** Participant characteristics by sleep condition.

Characteristic	Sleep restriction	Sleep extension	Total
Sample size, *n*	19	22	41
Continuous variables, mean (SD)			
Age, years	16.29 (1.28)	15.98 (1.04)	16.12 (1.15)
BMI	21.89 (2.27)	21.33 (2.01)	21.59 (2.13)
Categorical variables, *n* (%)			
Sex			
Male	8 (42.1%)	12 (54.5%)	20 (48.8%)
Female	11 (57.9%)	10 (45.5%)	21 (51.2%)
Pubertal maturation			
Pre-pubertal	1 (5.3%)	0 (0.0%)	1 (2.4%)
Mid-pubertal	3 (15.8%)	6 (27.3%)	9 (22.0%)
Late-pubertal	4 (21.1%)	6 (27.3%)	10 (24.4%)
Post-pubertal	11 (57.9%)	10 (45.5%)	21 (51.2%)

Participants were compensated based on study phase participation and estimated effort, with total compensation up to approximately CHF 500 (for detailed breakdown see Supplementary Table S1).

### Inclusion and exclusion criteria

Participants were healthy adolescents, with exclusions for sleep disorders, abnormal caffeine sensitivity, extreme chronotypes, substance use, or protocol non-compliance. All screening surveys were conducted in REDCap (Harris et al., 2009). Inclusion and exclusion criteria are provided in Supplementary Table S2.

### Study procedure

Participants were randomly assigned to SR (6 h time in bed) or SE [9.5 h time in bed, approximating adolescent sleep need (Short et al., 2018)] and completed two laboratory weeks in counterbalanced order, receiving caffeine 1 week and placebo the other, each following the two nights of the assigned sleep manipulation. This balanced ecological validity, ethical considerations, and feasibility. Each week comprised ambulatory sleep manipulation followed by laboratory testing. Participants abstained from caffeine throughout except for the blinded treatment; adherence to the sleep–wake schedule was verified by sleep diaries and actigraphy, and caffeine abstinence by salivary caffeine assay, with deviating participants excluded.

At the laboratory, participants completed the WOF before and after a blinded treatment of caffeine (2 mg/kg plus mannitol 2 mg/kg) or placebo (mannitol 2 mg/kg), with post-treatment assessment timed to peak caffeine levels. Testing was held at the same time of day across conditions; because sleep opportunity differed, the SR group was tested after longer wakefulness than the SE group. See Supplementary Data Sheet S1 for the study protocol and Supplementary Figure S1 for task timing.

### Randomisation of sleep manipulation and order of treatment

Participants were stratified by age (14–15.99, 16–17.99 years) and sex before randomisation. Sleep condition was assigned from a pre-generated slot list balanced across strata, with each enrolee allocated to the next available age- and sex-matched slot. Caffeine-placebo order was randomised by an external researcher keeping study researchers blinded.

### Measurement instrument

RDM was measured by the WOF (Blankenstein et al., 2017; Blankenstein et al., 2018) in E-prime 3.0.3.214 (Psychology Software Tools Inc, 2016). The WOF is a deliberative, value-based decision task in which risk is conveyed by explicitly displayed probabilities (shown as pie-chart stimuli) and reward magnitudes, in a non-social, non-affective context. Participants completed 57 trials drawn from 19 predefined trial types, comprising both safe-gamble and gamble-gamble trials with equal or unequal expected values (EV). On each trial they chose between 2 options within a 3,000 ms response window and received trial-by-trial outcome feedback; because probabilities and magnitudes were displayed but EV was not, participants had to integrate this information to evaluate the options. This trial structure, spanning safe-gamble and gamble-gamble trials at equal and unequal EV, yields the three outcome measures defined below.

RDM was evaluated using three continuous measures. Risk-taking propensity, the percentage of gambles chosen across trials with a safe alternative, captured overall willingness to gamble regardless of value, as in previous WOF studies (Blankenstein et al., 2017; Blankenstein et al., 2018). Optimal choice propensity, the percentage of higher-expected-value choices (including safe choices where a gamble had equal expected value), captured value sensitivity. Irrational choice propensity, the percentage of choices that were both gambles and non-optimal, captured value-insensitive risk-taking. Non-responses within the time limit were excluded (six participants, evenly distributed across sleep and drug conditions). Task visualisation, timing, and presentation are provided in Supplementary Figure S2, with trial-type specifications in Supplementary Table S3.

### Analysis

A hierarchical Bayesian framework analysed the effects of sleep and caffeine (Python, PyMC; ArviZ for summarisation and diagnostics). Outcomes were computed across many trials yielding an approximately continuous measure; therefore, they were expressed as proportions, standardised, and modelled with Gaussian likelihoods, to improve sampling stability and comparability across outcomes. Because Gaussian approximation is least tenable for irrational choice propensity, where some participants were near the floor, we refitted every model as a hierarchical binomial (logistic) model on the trial-level counts as a sensitivity analysis; the direction and practically negligible magnitude of all sleep, caffeine, and interaction effects were unchanged (Supplementary Data Sheet S2; Supplementary Table S4).

Each model included fixed effects for sleep condition, drug condition, their interaction, centred and standardised age, age category, and biological sex, with participant-level random intercepts to account for repeated measures. The primary estimands were the main effects of sleep condition and drug condition and their interaction. Weakly informative priors were specified for all parameters, and models were estimated using Hamiltonian Monte Carlo. Model convergence and sampling quality were assessed using standard diagnostic criteria. Full model specification, priors, preprocessing steps, and diagnostic procedures are reported in Supplementary Data Sheet S2.

## Results

Participant characteristics and baseline measures by sleep condition are presented in Tables 2, 3. Both manipulations were successful: sleep reduced under SR versus SE (time in bed 6.4 vs. 9.5 h; total sleep time 6.1 vs. 8.9 h; both *p* < 0.001), with subjective sleepiness (Karolinska Sleepiness Scale) rising under SR (change from baseline: morning +0.6 in SR vs. −0.8 in SE; evening +1.9 in SR vs. +0.1 in SE; both *p* < 0.001), and salivary caffeine, which before dosing did not differ between conditions (arrival: 0.21 vs. 0.14 μg/mL; *t*₄₀ = 1.68, *p* = 0.10) or between sleep groups (SR 0.20 vs. SE 0.16 μg/mL; *t* = 0.72, *p* = 0.48), was markedly elevated after caffeine versus placebo (1.37 vs. 0.09 μg/mL; *t*₄₀ = 7.03, *p* < 0.001).

**Table 3:** Baseline screening measures by sleep condition.

Characteristic	Sleep restriction, mean (SD)	Sleep extension, mean (SD)
Health and sleep measures		
Physical health (KIDSCREEN)	20.89 (1.85)	20.50 (2.11)
Psychological wellbeing (KIDSCREEN)	30.11 (2.13)	29.36 (4.02)
Subjective sleep quality (PSQI)	2.74 (1.28)	3.77 (1.23)
Daytime sleepiness (ESS)	6.21 (2.66)	4.50 (2.30)
Risk-taking, reward sensitivity, and socioeconomic measures		
Self-reported risk attitude	6.11 (1.94)	5.73 (1.75)
Brief sensation seeking scale total	25.95 (4.44)	25.64 (4.15)
BAS drive	11.26 (1.69)	12.41 (1.56)
BAS fun seeking	13.00 (1.80)	12.86 (1.70)
BAS reward responsiveness	14.05 (1.35)	13.95 (1.13)
BIS avoidance of unpleasantness	19.95 (4.67)	19.14 (2.90)
Monthly disposable money (CHF)	171.05 (174.35)	182.86 (306.54)

Values are presented as mean (SD). All measures were collected during screening or habituation prior to experimental manipulation.

**Figure 2:**
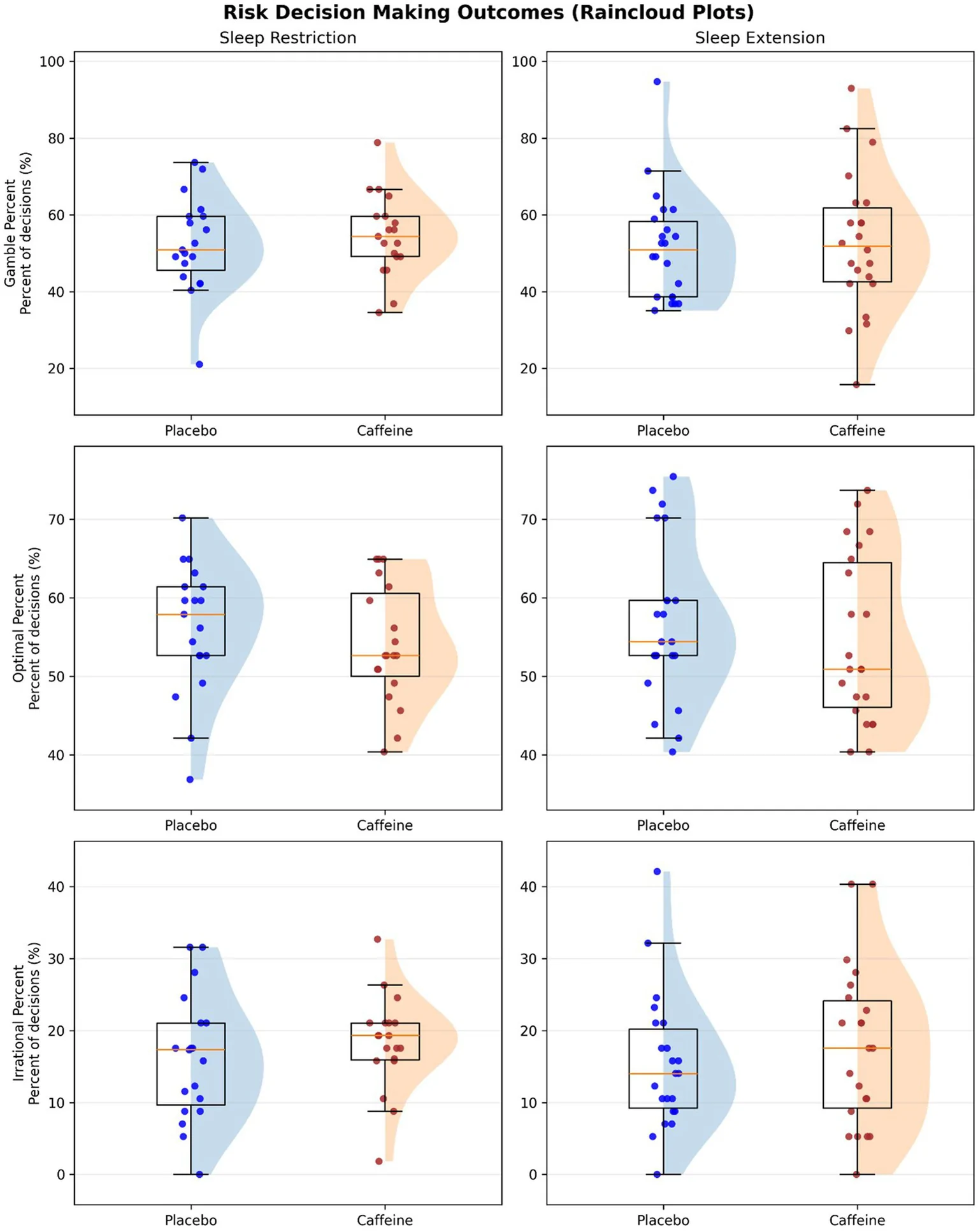
Risk decision making outcomes.

Groups were broadly comparable in demographic, developmental, health, sleep, and risk-related measures prior to experimental manipulation. Descriptive statistics for RDM outcomes by sleep and drug condition are shown in Table 4, and distributions of outcome variables across conditions are illustrated in Figure 2.

**Table 4:** Risk decision-making outcomes by sleep and drug condition.

Outcome	Sleep condition	Drug condition	*n*	Mean	SD	Median	Min	Max
Risk-taking propensity %	Sleep extension	Caffeine	22	52.79	18.16	51.75	15.79	92.98
		Placebo	22	51.46	14.26	50.88	35.09	94.74
	Sleep restriction	Caffeine	19	54.58	10.59	54.39	34.55	78.85
		Placebo	19	52.40	12.33	50.88	21.05	73.68
Optimal choice propensity %	Sleep extension	Caffeine	22	54.55	10.70	50.88	40.35	73.68
		Placebo	22	56.78	10.19	54.39	40.35	75.44
	Sleep restriction	Caffeine	19	54.02	7.55	52.63	40.35	64.91
		Placebo	19	56.14	8.27	57.89	36.84	70.18
Irrational choice propensity %	Sleep extension	Caffeine	22	17.62	11.17	17.54	0.00	40.35
		Placebo	22	15.43	9.42	14.04	0.00	42.11
	Sleep restriction	Caffeine	19	18.27	6.61	19.30	1.82	32.69
		Placebo	19	16.20	8.77	17.31	0.00	31.58

Values are descriptive statistics summarised by sleep and drug condition. *n* reflects the number of unique participants contributing data in each condition. Percentages reflect the proportion of trials per participant for each decision-making metric. Statistics are presented on the original (non-standardised) scale; standardisation was applied only for modelling.

All models showed good convergence and sampling quality, meeting standard diagnostic criteria (R-hat close to 1, large effective sample sizes, no divergent transitions). Initial divergences in one model were resolved by increasing the target acceptance rate without changing model structure or data inclusion (Supplementary Data Sheet S3). Parameter estimates are presented in Table 5. See Supplementary Figure S3 for posterior distributions of RDM outcomes.

**Table 5:** Hierarchical Bayesian regression estimates for risk decision-making outcomes.

Outcome	Predictor	Mean (*z*)	Mean (pp)	95% HDI (*z*)	95% HDI (pp)	*p* (direction)	BF01	ROPE (%)
Risk-taking propensity %	Intercept	0.023	–	[−0.448, 0.497]	–	–	–	–
	Age (centred, *z*-scored)	0.114	+1.59	[−0.226, 0.438]	[−3.16, +6.13]	0.75 (positive)	2.28	26.2
	Age group (young vs. old)	0.209	+2.93	[−0.412, 0.805]	[−5.77, +11.26]	0.75 (positive)	1.23	14.1
	Biological sex (male vs. female)	−0.144	−2.01	[−0.615, 0.303]	[−8.60, +4.25]	0.73 (negative)	1.78	20.2
	Drug condition (placebo vs. caffeine)	−0.089	−1.25	[−0.506, 0.321]	[−7.08, +4.49]	0.67 (negative)	2.20	24.6
	Sleep condition (SR vs. SE)	0.058	+0.80	[−0.444, 0.569]	[−6.21, +7.96]	0.59 (positive)	1.85	20.9
	Drug × Sleep interaction	−0.048	−0.67	[−0.600, 0.513]	[−8.39, +7.18]	0.57 (negative)	1.70	19.6
	Residual SD	0.859	–	[0.670, 1.072]	–	–	–	–
Optimal choice propensity %	Intercept	−0.365	–	[−0.814, 0.133]	–	–	–	–
	Age (centred, *z*-scored)	0.189	+1.74	[−0.143, 0.529]	[−1.32, +4.87]	0.87 (positive)	1.54	27.2
	Age group (young vs. old)	0.246	+2.27	[−0.365, 0.873]	[−3.36, +8.04]	0.78 (positive)	1.15	20.0
	Biological sex (male vs. female)	0.424	+3.90	[−0.048, 0.884]	[−0.44, +8.14]	0.96 (positive)	0.43	7.8
	Drug condition (placebo vs. caffeine)	0.205	+1.89	[−0.134, 0.564]	[−1.23, +5.20]	0.88 (positive)	1.46	25.5
	Sleep condition (SR vs. SE)	−0.066	−0.60	[−0.583, 0.426]	[−5.36, +3.92]	0.60 (negative)	1.90	32.4
	Drug × Sleep interaction	0.017	+0.16	[−0.470, 0.505]	[−4.32, +4.65]	0.53 (positive)	2.01	34.2
	Residual SD	0.694	–	[0.541, 0.862]	–	–	–	–
Irrational choice propensity %	Intercept	0.215	–	[−0.257, 0.688]	–	–	–	–
	Age (centred, *z*-scored)	0.035	+0.32	[−0.295, 0.355]	[−2.68, +3.23]	0.58 (positive)	2.91	48.0
	Age group (young vs. old)	0.098	+0.89	[−0.501, 0.715]	[−4.55, +6.49]	0.62 (positive)	1.51	26.2
	Biological sex (male vs. female)	−0.345	−3.13	[−0.802, 0.104]	[−7.28, +0.95]	0.93 (negative)	0.67	12.5
	Drug condition (placebo vs. caffeine)	−0.191	−1.73	[−0.592, 0.225]	[−5.37, +2.04]	0.83 (negative)	1.56	27.6
	Sleep condition (SR vs. SE)	0.036	+0.32	[−0.465, 0.543]	[−4.22, +4.93]	0.55 (positive)	1.90	33.0
	Drug × Sleep interaction	−0.023	−0.21	[−0.559, 0.532]	[−5.08, +4.83]	0.53 (negative)	1.85	31.4
	Residual SD	0.842	–	[0.659, 1.051]	–	–	–	–

Values are posterior means from hierarchical Bayesian models with participant-level random intercepts. Effects are reported in standardised units (*z*) and, in parentheses, converted to percentage points (pp) to aid interpretation. Positive coefficients indicate higher outcome values for males relative to females, placebo relative to caffeine, sleep restriction relative to sleep-extension, and younger relative to older participants. Because the model includes a drug-by-sleep interaction, each main effect is conditional on the reference level of the other factor: the sleep coefficient is the SR-SE difference under caffeine, and the drug coefficient is the placebo-caffeine difference under sleep-extension. The corresponding condition-specific contrasts, the sleep effect under placebo and the caffeine effect under each sleep condition, are reported in Supplementary Data Sheet S2 and Supplementary Table S5. 95% highest density intervals (HDIs) are shown in standardised units and percentage points. *p*(direction) denotes the posterior probability that the effect is positive or negative. BF₁₀ indicates Bayes factors comparing models with and without the corresponding parameter. Practical significance was assessed using a region of practical equivalence (ROPE) defined as ±1 percentage point in the original outcome scale; ROPE (%) indicates the proportion of posterior samples within this region. Residual SD refers to unexplained outcome variability. Standardisation and ROPE transformation were performed separately for each outcome variable. BF01 indicates Savage-Dickey Bayes factors for the null relative to the alternative; values above 1 favour the null, values below 1 favour an effect.

### Risk-taking propensity

For risk-taking propensity, posterior means for sleep condition, drug condition, and their interaction were close to zero, with 95% HDIs overlapping zero and non-trivial ROPE overlap (Table 5). Bayes factors (BFs) indicated only small shifts relative to the null (drug condition BF01 = 2.20; sleep condition BF01 = 1.85; interaction BF01 = 1.70), providing limited evidence in either direction. Age showed a weak positive directional tendency (*p*(direction) = 0.75), while age group and biological sex were uncertain with intervals spanning zero.

### Optimal choice propensity

For optimal choice propensity, posterior means for sleep condition and the drug × sleep interaction were near zero, with 95% HDIs overlapping zero and substantial ROPE overlap (Table 5). Biological sex showed a positive mean (+3.90 pp) and drug condition a small difference favouring placebo (+1.89 pp), but both intervals spanned zero with substantial posterior mass within the ROPE. Age and age group showed positive directional tendencies with intervals overlapping zero. Bayes factors were mixed and mostly small-to-moderate, indicating limited evidence for robust effects beyond the sex difference.

### Irrational choice propensity

For irrational choice propensity, posterior means for sleep condition, drug condition, and their interaction were close to zero, with 95% HDIs overlapping zero and substantial ROPE overlap (Table 5). Bayes factors were generally small-to-moderate. Age and age group showed weak positive directional tendencies and biological sex a negative mean (−3.13 pp), but all 95% HDIs overlapped zero.

To clarify the independent and combined effects of the two manipulations, condition-specific contrasts are reported in full in Supplementary Data Sheet S2 (Supplementary Table S5). Under placebo, SR had negligible effects on all outcomes, and the caffeine effects were small and similar under both sleep conditions; the drug-by-sleep interaction was negligible throughout (at most 0.67 pp) and every 95% HDI spanned zero. The largest single contrast, the caffeine effect on optimal choice, remained uncertain, with its 95% HDI spanning zero and substantial posterior mass within the ROPE.

These conclusions were robust to the modelling of the outcomes: a hierarchical binomial sensitivity analysis on the trial-level counts reproduced the negligible sleep, caffeine, and interaction effects across all 3 outcomes (Supplementary Data Sheet S2; Supplementary Table S4).

## Discussion

Using a mixed experimental design with within-subject, double-blind caffeine manipulation and between-subject sleep manipulation, this study examined independent and interactive effects of two nights of SR and acute caffeine on adolescent RDM. Contrary to preregistered hypotheses, neither manipulation produced robust or practically meaningful changes in risk-taking, optimal choice, or irrational choice propensity, and the sleep-caffeine interaction was not credibly different from zero. Together, these findings provide no clear evidence for practically meaningful average effects of two nights of SR, acute caffeine, or their interaction on value-based RDM under the present task and protocol. We interpret these null effects across all 3 outcomes primarily in terms of task sensitivity. The modified WOF captures deliberative, value-based choice from explicitly displayed probabilities and reward magnitudes, and prior work indicates it engages valuation and cognitive control systems and captures individual differences in risk and ambiguity processing (Blankenstein et al., 2017; Blankenstein et al., 2018). Unlike escalating-risk tasks such as the BART, probability-learning or ambiguity-based paradigms, and affectively charged or peer-influenced contexts that often drive adolescent risk behaviour, the WOF presents static, non-escalating risk in a non-social context, and such deliberative choices may be less responsive to short-term sleep and arousal manipulations than more dynamic, affective, or socially salient forms of risk-taking. On this reading, the null effects delineate a boundary condition of this paradigm rather than indicating that SR or caffeine leaves adolescent risk behaviour unaffected (Lim et al., 2025).

These findings contrast with epidemiological evidence linking insufficient sleep to adolescent real-world risk behaviour (Short and Weber, 2018) and experimental findings from Davis et al. (2013), showing increased risk-taking following SR in a virtual pedestrian crossing task. Such discrepancies likely reflect differences in task demands and context (Fu et al., 2026; Wei et al., 2025). Although ecologically relevant, the SR protocol may have been insufficient in duration or severity to shift decision-making beyond ROPEs on this modified WOF. Moreover, heterogeneity in adolescent sleep duration, caffeine exposure, and resilience to SR may have attenuated average effects despite meaningful individual sensitivity (Galli et al., 2022; Salfi et al., 2020).

Caffeine effects also differed from Temple et al. (2017), who observed dose- and habit-dependent effects on adolescent risk-taking using the BART. Methodological factors may explain this discrepancy. The BART emphasises learning under uncertainty and escalating risk, whereas the WOF isolates value-based choice under explicit probabilities and reward magnitudes, requiring participants to integrate this information to estimate expected value, and the sample was restricted to low or non-habitual caffeine users, so the moderate- and high-use subgroups in which such effects emerge were not represented. The 2 mg/kg caffeine dose remained within established safety margins, minimising potential adverse effects (Reichert et al., 2021; Bonnar and Gradisar, 2015), but though expected to induce arousal and alertness, may have been insufficient to affect decision-making in this task. Higher doses may be required to detect group-level effects in samples heterogeneous in habitual consumption.

Neurodevelopmental models emphasise a maturational imbalance in which heightened subcortical reward sensitivity coincides with slower-developing prefrontal control (van Duijvenvoorde et al., 2016; Van Leijenhorst et al., 2010; Arain et al., 2013). Within this framework, adolescent value-based decision-making is shaped primarily by longer-term prefrontal-striatal trajectories rather than transient physiological state and the present findings are consistent with this: two nights of SR and acute caffeine exerted limited influence on the integration of valuation and cognitive control. Such transient changes in arousal, alertness, and cortical excitability (Diukova et al., 2012) may not translate into measurable behavioural shifts within still-developing neural systems. Age- and sex-related covariates showed directional tendencies for some outcomes, but estimates were imprecise and all uncertainty intervals overlapped zero and are best considered exploratory.

Although the mean proportion of gamble choices was close to 50%, this does not indicate guessing. The WOF includes trial types in which gambling is optimal on some trials but suboptimal on others, such that value-sensitive responding requires both gambling and safe choices. Consistent with this, participants showed substantial interindividual variability and a marked dissociation between optimal (≈55%) and irrational (≈16%) choices (Table 4), rather than the elevated irrational choice rates expected under indiscriminate responding.

Testing followed different durations of wakefulness in the SR (16 h) and SE (12 h) conditions, so effects reflect a combined sleep–wake manipulation rather than sleep alone. Assessments occurred during the circadian phase associated with elevated alertness (Dijk et al., 1992), which may have reduced sensitivity to sleep-related differences, particularly under SE, and should be considered when interpreting the small and null effects. Alternative designs separating wake duration from circadian phase would introduce further confounds, such as practice effects, and were not feasible.

Hierarchical Bayesian models enabled explicit quantification of uncertainty and practical equivalence. Directionally consistent estimates were accompanied by substantial posterior uncertainty, underscoring the importance of distinguishing statistical credibility from practical relevance in adolescent behavioural research.

This study has several methodological strengths. It employed a mixed design combining within-subject, counterbalanced, double-blind caffeine manipulation with between-subject sleep manipulation and rigorous adherence monitoring. Participants were screened to exclude confounds related to caffeine use, sleep disturbances, extreme chronotypes, and recent circadian disruption.

Several limitations warrant consideration. Although sleep–wake and abstinence adherence was verified and enforced by exclusion, achieved sleep was quantified only via self-report; weaker-than-intended sleep manipulation therefore cannot be fully excluded as a contributor to the null effects. Although sample size was adequate for hierarchical Bayesian estimation, precision for detecting small interaction effects, particularly sleep-caffeine interactions, may have been limited. The conservative caffeine dose may have constrained the magnitude of observable effects. The WOF may not generalise to socially or emotionally salient contexts characteristic of adolescence. The absence of robust effects should not be interpreted as evidence that SR or caffeine has no impact; rather, such effects were not reliably detected within the constraints of the present task, dose, and design.

Future research should examine a broader range of caffeine doses and extend the duration or severity of SR to determine whether stronger or more sustained perturbations influence adolescent RDM. Tasks that probe socially and emotionally salient risk may clarify the boundary conditions of the present findings. Additionally, combining behavioural modelling with neuroimaging measures, such as fMRI or EEG, may reveal neural markers of valuation, cognitive control, or reward sensitivity even in the absence of behavioural change (Lim et al., 2025). Finally, larger samples would improve precision and support mixture models to identify subgroups or responder phenotypes with differential sensitivity to SR and caffeine.

## Conclusion

Under the specific conditions of this study (two nights sleep manipulation, 2 mg/kg caffeine dose, adolescents aged 14–17, and a modified WOF task), we found no clear evidence of practically meaningful average changes in RDM, and these findings do not support caffeine as a compensatory strategy for short-term SR in adolescents. This should not be read as evidence that such perturbations are inconsequential; rather it highlights the need for future research to identify boundary conditions, dose–response relationships, and individual differences that may confer greater sensitivity.

## Data Availability

The raw data supporting the conclusions of this article will be made available by the authors, without undue reservation.

## Footnotes

1 https://osf.io/7fvyw.
