# Supplementary material for: Limited change in adolescent value-based risk choices following acute sleep restriction and caffeine
Source: Front Psychol. 2026 Aug 12;17:1879309. doi: 10.3389/fpsyg.2026.1879309 (PMC13506745; doi:10.3389/fpsyg.2026.1879309)
Supplement: Supplementary file 1 [file Data_Sheet_1.docx]

**Supplementary Data Sheet S1** | Study procedure

Participants completed two study weeks in a counterbalanced order, receiving both caffeine and placebo while assigned to either sleep restriction (SR; 6 h time in bed) or sleep extension (SE; 9.5 h time in bed), followed by laboratory testing (see Supplementary Figure S1 for further detail). The two study weeks were performed within a three-month period with no minimum period between study weeks. Throughout the study, participants were instructed to abstain from caffeine except for the blinded treatment administered during the laboratory phase. Each study week comprised an ambulatory phase and a laboratory phase. During the ambulatory phase, participants first completed a three-day stabilisation period with an 8-16 h sleep-wake schedule (±60 min) to minimise the influence of historical sleep debt. Following stabilisation, participants adhered to the assigned SR or SE schedule for two consecutive nights (±30 min). The six-hour SR duration was selected based on evidence demonstrating neurobehavioral effects of sleep restriction and prior experimental paradigms in adolescents (Banks and Dinges, 2007; Davis et al., 2013; de Bruin et al., 2017), whereas the 9.5-hour SE duration provided an extended sleep opportunity within recommended adolescent sleep ranges (Carskadon, 2011; Jenni et al., 2005; Short et al., 2018). Sleep schedules were aligned to each participant’s midpoint of sleep on free days as determined by the Munich Chronotype Questionnaire.

Following the ambulatory phase, participants reported to the Centre for Chronobiology approximately 12 h 10 min after waking in the SR condition and 8 h 40 min after waking in the SE condition. The Wheel of Fortune (WOF) task was administered approximately 14 h after waking in SR and 10 h after waking in SE. One hour after the first WOF, participants received a blinded treatment of either caffeine (2 mg/kg) or placebo. Caffeine and placebo capsules were identical in appearance and contained mannitol as an inert filler, with caffeine capsules additionally containing caffeine (2 mg/kg). All treatments were encapsulated in two opaque capsules manufactured by Kapselwelt (Hude, Germany) using compounds produced by Hänseler AG (Herisau, Switzerland). Forty-five minutes after treatment administration, scheduled to coincide with peak caffeine levels, participants completed a second WOF. Task scheduling and execution during the laboratory phase were managed using PsyTestPro (Mattern, Capdevila and Lane), an open-source Python application developed for use in sleep research and available at https://github.com/eliasmattern/PsyTestPro.

The timing of post-treatment testing was designed to capture the effects of prolonged wakefulness under SR while maintaining comparability across conditions in circadian phase relative to wake time and in interval between treatment administration and testing. Under SR, post-treatment assessment occurred approximately sixteen hours after waking, whereas under SE it occurred approximately twelve hours after waking. Circadian phase alignment was prioritised over wake-duration equivalence, resulting in differences in wake duration that should be interpreted in the context of the sleep manipulation rather than circadian misalignment. Given participants’ young age and limited prior caffeine exposure, a conservative caffeine dose was selected relative to adult studies. Saliva samples and Karolinska Sleepiness Scale (KSS) ratings were collected at regular 50-minute intervals during the laboratory phase, the saliva to verify caffeine abstinence before treatment and monitor caffeine levels after it. Time in bed and total sleep time were derived from a study-specific daily sleep diary, averaged over the two restriction nights; wrist actigraphy served as an on-arrival adherence check only. KSS was additionally recorded each morning and evening in the diary, and manipulation checks used these diary ratings. Participants also completed additional questionnaires, cognitive tasks, and neuroimaging assessments (EEG and MRI) as part of the broader study protocol; these measures are not reported in the present manuscript.

**References**

Banks, S., and Dinges, D. F. (2007). Behavioral and physiological consequences of sleep restriction. J. Clin. Sleep Med. 3, 519–528. doi: 10.5664/jcsm.26918

Carskadon, M. A. (2011). Sleep in adolescents: the perfect storm. Pediatr. Clin. North Am. 58, 637–647. doi: 10.1016/j.pcl.2011.03.003

Davis, A. L., Avis, K. T., and Schwebel, D. C. (2013). The effects of acute sleep restriction on adolescents’ pedestrian safety in a virtual environment. J. Adolesc. Health 53, 785–790. doi: 10.1016/J.JADOHEALTH.2013.07.008

de Bruin, E. J., van Run, C., Staaks, J., and Meijer, A. M. (2017). Effects of sleep manipulation on cognitive functioning of adolescents: a systematic review. Sleep Med. Rev. 32, 45–57. doi: 10.1016/j.smrv.2016.02.006

Jenni, O. G., Achermann, P., and Carskadon, M. A. (2005). Homeostatic sleep regulation in adolescents. Sleep 28, 1446–1454. doi: 10.1093/SLEEP/28.11.1446

Short, M. A., Weber, N., Reynolds, C., Coussens, S., and Carskadon, M. A. (2018). Estimating adolescent sleep need using dose-response modeling. Sleep 41, 1–14. doi: 10.1093/SLEEP/ZSY011
