# Supplementary material for: Limited change in adolescent value-based risk choices following acute sleep restriction and caffeine
Source: Front Psychol. 2026 Aug 12;17:1879309. doi: 10.3389/fpsyg.2026.1879309 (PMC13506745; doi:10.3389/fpsyg.2026.1879309)
Supplement: Supplementary file 2 [file Data_Sheet_2.docx]

**Supplementary Data Sheet S2** | Hierarchical Bayesian statistical analysis

Analyses were conducted using a hierarchical Bayesian framework implemented in Python. Hierarchical Bayesian regression models with participant-level random intercepts were specified using the PyMC package, and ArviZ was used for posterior summarisation, visualisation, and diagnostic checks. A Bayesian approach was selected because it provides full posterior distributions for parameters rather than relying solely on asymptotic p-values, supports direct probability statements about effect direction and magnitude, and is well suited to multilevel data structures where repeated observations are nested within participants. The use of PyMC and ArviZ facilitates transparent, reproducible modelling with modern Hamiltonian Monte Carlo sampling and standard diagnostics.

Separate models were fit for three continuous risk decision-making outcome variables derived from an adapted version of the Wheel of Fortune task: risk-taking propensity, optimal choice propensity, and irrational choice propensity. Outcomes were modelled separately to avoid imposing constraints that would be required by a multivariate outcome model and to keep interpretation straightforward for each behavioural endpoint. Each model included fixed effects for sleep condition (sleep restriction vs sleep extension), drug condition (caffeine vs placebo), their interaction, centred and standardised age (age_centred_z), age group, and biological sex. The primary estimands were the main effects of sleep condition and drug condition and their interaction, because the interaction directly tests whether caffeine effects differ by sleep condition, which is the theoretically relevant moderation hypothesis. Random intercepts were included for participants to account for repeated measures and individual-level variability.

Stratification variables used during randomisation were retained in the models to ensure robust inference and to account for potential residual imbalance across conditions. Although stratified randomisation is designed to balance groups, finite samples can still exhibit chance imbalance within strata. Retaining stratification variables protects against biased estimation driven by residual imbalance and ensures that inferences remain aligned with the experimental design. Inclusion of the caffeine-by-sleep condition interaction term allowed assessment of whether caffeine effects differed between sleep restriction and sleep extension conditions, which addresses a plausible physiological and behavioural mechanism and prevents misinterpretation of an average main effect that could mask condition-specific effects.

Outcome variables were expressed as proportions and standardised prior to modelling. Percentage values were converted to proportions by dividing by 100, which places the outcomes on a consistent 0 to 1 scale and avoids potential confusion arising from arbitrary percentage scaling. The proportion variables were then z-transformed using the mean and population standard deviation of each variable, resulting in standardised outcomes with mean zero and unit variance. Outcome standardisation was used for three reasons. First, it improves numerical stability for Hamiltonian Monte Carlo sampling and reduces posterior geometry pathologies that can occur when predictors or outcomes are on very different scales. Second, it makes regression coefficients comparable across the three outcomes, facilitating interpretation of relative effect sizes. Third, it allows the use of weakly informative priors specified in standard deviation units that are coherent across outcomes. These standardised outcome variables were used as dependent variables in the hierarchical Bayesian analyses.

Each standardised outcome was modelled with a Gaussian (normal) likelihood. Although the underlying variables are proportions, a normal approximation is reasonable here: each proportion was computed across a substantial number of trials within the 57-trial task, yielding a finely graded, approximately continuous measure rather than a coarse count, and for risk-taking propensity and optimal choice propensity the observed values clustered around the centre of the scale (condition means of approximately 51-55% and 54-57%, respectively; main-text Table 4), well away from the 0 and 1 boundaries at which a Gaussian likelihood is least tenable. Irrational choice propensity was lower on average (condition means of approximately 15-18%) with a small number of participants at the floor, so the normal approximation is most strained for this outcome. To confirm that the substantive conclusions did not depend on this distributional choice, a sensitivity analysis was conducted using a hierarchical binomial (logistic) model fitted to the underlying trial-level counts, retaining the same fixed-effect and random-intercept structure, as reported below.

Each outcome was refitted with a hierarchical binomial model, in which the number of qualifying choices was treated as a binomial count out of the number of qualifying trials per participant and condition (logit link), retaining the same fixed effects, participant-level random intercept, sampler, and convergence criteria as the primary analysis. Coefficient priors were Normal(0, 0.5) on the logit scale, the intercept prior was Normal(0, 1), and the participant random-intercept standard deviation was assigned a HalfNormal(0.5) prior; because the binomial likelihood has no residual dispersion term, the HalfNormal(1.0) residual prior used in the Gaussian model does not apply. Because logistic coefficients are on the log-odds scale, each effect was summarised as an average marginal effect in percentage points, computed by predicting the choice probability at each level of the predictor across the observed sample and taking the difference, and was evaluated against the same ±1 percentage point region of practical equivalence (ROPE). This model also accommodates the bounded, count-based nature of the outcomes directly, including the participant-specific trial totals produced by excluded timed-out trials and the floor in irrational choice propensity.

**Supplementary Table S4: Gaussian vs binomial comparison.** Comparison of the primary standardised Gaussian model and the binomial sensitivity model for the sleep, drug, and interaction effects. Effects are posterior means in percentage points (pp); for the binomial model these are average marginal effects. ROPE (%) is the proportion of the posterior within ±1 percentage point.

| **Outcome** | **Predictor** | **Primary (pp)** | **Primary ROPE%** | **Binomial (pp)** | **Binomial ROPE%** |
| --- | --- | --- | --- | --- | --- |
| Risk-taking propensity | Sleep (SR vs SE) | +0.80 | 20.9 | +0.24 | 19.3 |
|  | Drug (placebo vs caffeine) | -1.25 | 24.6 | -1.36 | 31.1 |
|  | Drug x Sleep | -0.67 | 19.6 | -0.77 | 27.6 |
| Optimal choice propensity | Sleep (SR vs SE) | -0.60 | 32.4 | -0.68 | 27.1 |
|  | Drug (placebo vs caffeine) | +1.89 | 25.5 | +2.19 | 22.2 |
|  | Drug x Sleep | +0.16 | 34.2 | -0.08 | 27.8 |
| Irrational choice propensity | Sleep (SR vs SE) | +0.32 | 33.0 | +0.47 | 31.2 |
|  | Drug (placebo vs caffeine) | -1.73 | 27.6 | -2.20 | 18.2 |
|  | Drug x Sleep | -0.21 | 31.4 | +0.25 | 37.7 |

Across all three outcomes, the binomial model reproduced the direction and the practically negligible magnitude of the sleep, drug, and interaction effects: every 95% highest density interval continued to span zero, the average marginal effects remained within or close to the ±1 percentage point region, and the covariate pattern was unchanged, including the comparatively larger effect of biological sex, which again showed the smallest ROPE overlap. The substantive conclusions were therefore not dependent on the standardised Gaussian modelling choice.

To make the independent and combined effects of sleep restriction and caffeine explicit, the model-implied condition-specific contrasts were computed directly from the posterior samples as linear combinations of the sleep, drug, and interaction parameters and converted to percentage points.

**Supplementary Table S5: condition-specific contrasts.** Condition-specific contrasts derived from the hierarchical Bayesian models. Posterior mean differences are in percentage points (pp), with 95% HDIs, posterior probability of direction, and the proportion of the posterior within the ±1 pp ROPE. Positive values denote a higher outcome for the first-named condition (sleep restriction relative to sleep extension; caffeine relative to placebo).

| **Outcome** | **Contrast** | **Mean (pp)** | **95% HDI (pp)** | **P(direction)** | **ROPE (%)** |
| --- | --- | --- | --- | --- | --- |
| Risk-taking | SR-Placebo vs. SE-Placebo | +0.14 | [-7.96, +8.13] | 0.51 (positive) | 19.4 |
|  | SE-Caffeine vs. SE-Placebo | +1.25 | [-4.49, +7.08] | 0.67 (positive) | 24.6 |
|  | SR-Caffeine vs. SR-Placebo | +1.91 | [-4.99, +8.53] | 0.72 (positive) | 19.8 |
|  | Drug × Sleep interaction | -0.67 | [-8.39, +7.18] | 0.57 (negative) | 19.6 |
| Optimal choice | SR-Placebo vs. SE-Placebo | -0.45 | [-5.56, +4.72] | 0.56 (negative) | 29.8 |
|  | SE-Caffeine vs. SE-Placebo | -1.89 | [-5.20, +1.23] | 0.88 (negative) | 25.5 |
|  | SR-Caffeine vs. SR-Placebo | -2.05 | [-5.76, +1.77] | 0.86 (negative) | 23.1 |
|  | Drug × Sleep interaction | +0.16 | [-4.32, +4.65] | 0.53 (positive) | 34.2 |
| Irrational choice | SR-Placebo vs. SE-Placebo | +0.12 | [-5.08, +5.24] | 0.52 (positive) | 29.7 |
|  | SE-Caffeine vs. SE-Placebo | +1.73 | [-2.04, +5.37] | 0.83 (positive) | 27.6 |
|  | SR-Caffeine vs. SR-Placebo | +1.94 | [-2.40, +6.22] | 0.82 (positive) | 24.1 |
|  | Drug × Sleep interaction | -0.21 | [-5.08, +4.83] | 0.53 (negative) | 31.4 |

Every contrast was small and each 95% HDI spanned zero; the within-condition caffeine effects were close to one another, consistent with the near-zero interaction.

Age was represented using a combination of a categorical and a continuous component to preserve the logic of stratified randomisation while retaining information about continuous age-related variation. Chronological age was centred at the stratification boundary (16 years) and then standardised to yield age_centred_z, such that one unit corresponded to one standard deviation of age in the sample (approximately 1.14 years). This parameterisation was chosen to address two common reviewer concerns: loss of information from dichotomising a continuous variable and collinearity when combining group membership with a raw continuous age measure. Including both age category and centred continuous age allows the model to estimate a group contrast at the stratification boundary while also capturing linear age-related variation across the full sample. Centring age at the boundary reduces redundancy between the categorical and continuous age terms, mitigating collinearity and yielding coefficients with clear interpretation. Under this parameterisation, the age category coefficient reflects the difference between younger and older participants evaluated at age 16, and the age_centred_z coefficient reflects the linear association between age and outcome across participants per approximately 1.14 years. This approach respects the stratified design while preserving within-range developmental information that would be discarded by modelling only the age category.

Robust outlier detection was performed within subgroups defined by sex, age category, drug condition, and sleep condition. For each outcome, robust z scores were computed using subgroup-specific medians and median absolute deviations (MAD), rescaled to standard deviation units. Subgroup-specific robust scaling was chosen because pooling across experimental and demographic subgroups can incorrectly label valid condition-dependent values as outliers if groups differ in central tendency or dispersion. Using the median and MAD reduces sensitivity to extreme values compared with mean and standard deviation based approaches. Observations exceeding ±3 robust z units were identified as potential outliers and examined descriptively, including visual inspection of distributions. No observations were excluded based on this procedure. This decision was made to avoid researcher degrees of freedom associated with selective exclusion and to ensure that inference reflects the full observed data. The outlier inspection served as a diagnostic check to detect data entry errors, impossible values, or gross anomalies, rather than as a basis for data-dependent exclusion.

Categorical predictors were coded as binary variables for inclusion in the models. Sex was coded as 0 = female and 1 = male; drug condition was coded as 0 = caffeine and 1 = placebo; sleep condition was coded as 0 = sleep extension (SE) and 1 = sleep restriction (SR); and age category was coded as 0 = older and 1 = younger. This coding supports transparent interpretation of regression coefficients as conditional mean differences between coded groups. Under this coding scheme, positive regression coefficients indicate higher outcome values for males relative to females, placebo relative to caffeine, sleep restriction relative to sleep extension, and younger relative to older participants, holding other predictors constant. Making the reference categories explicit is essential for reproducibility and prevents ambiguity in interpreting main effects and interactions.

Model parameters were assigned weakly informative priors appropriate for standardised outcomes. The intercept was assigned a Normal (0, 1) prior, reflecting the expectation that, on a standardised outcome scale, baseline performance is likely to fall within a few standard deviations of zero while allowing sufficient flexibility for shifts in the intercept due to covariate coding and partial pooling. Fixed-effect coefficients were assigned Normal(0, 0.5) priors to regularise estimates toward small effects while allowing substantively meaningful deviations. This is a deliberate guard against overfitting and unstable estimates in models with multiple predictors and an interaction, especially when effects are expected to be modest in behavioural data. Participant-level random intercept variability was modelled with a HalfNormal (0.5) prior, which regularises individual-difference variance toward plausible values while allowing substantial between-participant heterogeneity when supported by the data. Residual variability was modelled with a HalfNormal (1.0) prior, which is weakly informative on the standardised scale and accommodates a wide range of noise levels without encouraging implausibly large values. Models were fit using Hamiltonian Monte Carlo with the No-U-Turn Sampler because it is efficient for continuous-parameter hierarchical models and typically mixes better than random-walk samplers. For each outcome, four chains were run with 10,000 tuning iterations and 6,000 posterior draws per chain to obtain stable posterior summaries and reliable diagnostics. Convergence and sampling quality were assessed using the maximum R-hat across parameters, the minimum effective sample size, and the absence of divergent transitions. Divergences were treated as a critical indicator of problematic posterior geometry and potential bias in estimates. If diagnostic criteria were not met, the target acceptance rate was increased and the model refit, up to a maximum of three attempts, to reduce step size and mitigate divergences while preserving the model specification. This procedure makes the sampling strategy explicit and limits adaptive researcher discretion.

To support interpretation in the original behavioural metric, parameter estimates were reported both in standardised units and in percentage points. Although models were fit to standardised outcomes, posterior samples of each fixed-effect coefficient were converted to the original scale to communicate effect sizes in units that are directly meaningful for decision behaviour. Conversion to percentage points was performed by multiplying posterior coefficient samples by the empirical standard deviation of the corresponding raw outcome expressed as a proportion (0–1), and then multiplying by 100. This produces an estimated change in percentage points associated with a one-unit change in the predictor, holding other predictors constant. Practical significance was assessed using a region of practical equivalence (ROPE) defined as ±1 percentage point in the original scale. This threshold was chosen to reflect a small effect that is unlikely to be of substantive behavioural relevance while remaining sensitive to meaningful changes in decision-making. Defining ROPE in the original metric improves interpretability and reduces arbitrariness relative to specifying ROPE only in standard deviation units. For each outcome, the ±1 percentage point ROPE was transformed into the corresponding interval in standardised units using the empirical standard deviation of that outcome, and the proportion of posterior samples falling within the ROPE was computed. Reporting the posterior mass within the ROPE provides an explicit measure of evidence that an effect is practically negligible, complementing uncertainty intervals and directional probabilities.

Evidence for the null hypothesis of no effect was additionally summarised using Bayes factors (BF01). Bayes factors were computed using the Savage–Dickey density ratio comparing posterior density at zero with the corresponding prior density, consistent with the Normal (0, 0.5) prior specified for regression coefficients. Posterior density was estimated using kernel density estimation over posterior draws. Values greater than one indicate evidence favouring the null, whereas values less than one indicate evidence favouring the presence of an effect relative to the null. Bayes factors were interpreted descriptively alongside credible intervals, directional probabilities, and ROPE proportions rather than as stand-alone decision criteria.

All model results, numerical summaries, and diagnostic figures were compiled into a structured report document, including convergence metrics, posterior summaries, trace plots, and posterior distributions for regression coefficients. This reporting pipeline was implemented to ensure reproducibility and transparency by pairing numerical convergence criteria with visual diagnostics and by presenting posterior results in both standardised and original units to support substantive interpretation. Automating reporting reduces the risk of selective reporting, ensures consistent summaries across outcomes, and provides a complete audit trail linking model specification, diagnostics, and reported results.
