# Supplementary material for: Limited change in adolescent value-based risk choices following acute sleep restriction and caffeine
Source: Front Psychol. 2026 Aug 12;17:1879309. doi: 10.3389/fpsyg.2026.1879309 (PMC13506745; doi:10.3389/fpsyg.2026.1879309)
Supplement: Supplementary file 3 [file Data_Sheet_3.docx]

**Supplementary Data Sheet S3 | Markov Chain Monte Carlo diagnostics and sampling robustness**

All hierarchical Bayesian models were estimated using Hamiltonian Monte Carlo with the No-U-Turn Sampler. For each outcome, four independent Markov chains were run with 10,000 tuning iterations and 6,000 posterior draws per chain. Sampling diagnostics were evaluated using multiple complementary criteria, including the potential scale reduction factor (R-hat), effective sample sizes (ESS), and the presence of divergent transitions.

For the risk-taking propensity outcome, the initial model fit exhibited a small number of divergent transitions (6 divergences). To address this, the target acceptance rate was increased and the model refit. The final model showed no divergent transitions, with a maximum R-hat of approximately 1.001 and a minimum effective sample size exceeding 1,900.

For the optimal choice propensity outcome, the initial model fit converged without divergences. The maximum R-hat was approximately 1.001, and effective sample sizes were high across parameters, with minimum effective sample size exceeding 7,000.

For the irrational choice propensity outcome, the initial model fit converged without divergent transitions. The maximum R-hat was approximately 1.001 and minimum effective sample sizes exceeded 3,600 across parameters.

Across all outcomes, final models satisfied standard convergence criteria, including R-hat values close to 1, large effective sample sizes, and the absence of divergent transitions. Trace plots and posterior density plots were inspected to confirm adequate chain mixing and stable posterior distributions. Importantly, no model terms were added or removed and no data were excluded during the adaptation process, ensuring that convergence improvements did not reflect post hoc model modification.

Taken together, these diagnostics indicate that posterior estimates are unlikely to be driven by sampling artefacts and can be interpreted with confidence.

**Table S3.1.** **Summary of convergence diagnostics for hierarchical Bayesian models.**
Initial divergences refer to the first model fit before increasing the target acceptance rate. Final diagnostics correspond to the accepted model used for inference.

| **Outcome variable** | **Max R-hat** | **Min ESS** | **Divergences (initial)** | **Divergences (final)** | **Chains** | **Tuning iterations** | **Posterior draws per chain** |
| --- | --- | --- | --- | --- | --- | --- | --- |
| Risk-taking propensity | 1.001 | 1,965 | 6 | 0 | 4 | 10,000 | 6,000 |
| Optimal choice propensity | 1.001 | 7,352 | 0 | 0 | 4 | 10,000 | 6,000 |
| Irrational choice propensity | 1.001 | 3,650 | 0 | 0 | 4 | 10,000 | 6,000 |

As shown in Table S3.1, all final models satisfied standard convergence criteria, including R-hat values close to 1, large effective sample sizes, and the absence of divergent transitions. Initial divergences observed in one model were resolved by increasing the target acceptance rate, without altering model structure or data inclusion. These results indicate stable posterior estimation and reliable inference across all outcomes.
