# Supplementary material for: Limited change in adolescent value-based risk choices following acute sleep restriction and caffeine
Source: Front Psychol. 2026 Aug 12;17:1879309. doi: 10.3389/fpsyg.2026.1879309 (PMC13506745; doi:10.3389/fpsyg.2026.1879309)
Supplement: Supplementary file 4 [file Supplementary_file_1.docx]

**Supplementary Figure S1** | Overview of task timing

The Wheel of Fortune task was administered twice on day two (D2), once before and once after blinded caffeine or placebo administration. Analyses in this manuscript focus on performance during the second Wheel of Fortune session.

Additional cognitive tasks (e.g., inhibition and vigilance measures) and questionnaires (e.g., subjective sleepiness ratings) were also collected. Further, saliva samples were regularly taken to determine caffeine and melatonin levels. Analyses of these data are beyond the scope of the present manuscript.
