# Supplementary material for: Limited change in adolescent value-based risk choices following acute sleep restriction and caffeine
Source: Front Psychol. 2026 Aug 12;17:1879309. doi: 10.3389/fpsyg.2026.1879309 (PMC13506745; doi:10.3389/fpsyg.2026.1879309)
Supplement: Supplementary file 5 [file Supplementary_file_2.docx]

**Supplementary Figure S2** | Wheel of Fortune task structure and visualisation

**Figure S2.** Wheel of Fortune Task Visualisation

Participants completed 57 trials comprising safe-gamble and gamble-gamble trials, which included both equal and unequal expected value (EV) comparisons.

Each trial began with a fixation cross displayed for 500 ms. Two choice options were then presented, one on the left and another on the right side of the screen, with option position randomized across trials. Participants had up to 3000 ms to indicate their choice. Following a response, the selected option was highlighted by a white square for 750 ms. Outcome feedback was then presented for 1500 ms and consisted of a smiley or sad face accompanied by the outcome value (win amount or zero). If no response was made within the response window, the message “TOO LATE” was displayed for 1000 ms. Each trial ended with a fixation cross presented for 1250 ms before the onset of the subsequent trial.

Probability information was conveyed using circular pie chart stimuli. Safe options were displayed as fully white circles, indicating a guaranteed outcome. Gamble options were displayed as partially white circles, with the proportion of the white segment representing the probability of winning. For each gamble type, multiple image variants were used that differed only in the orientation of the white segment, while the represented probability remained constant across variants. The represented probabilities corresponded directly to the expected values defined by the stimulus sampling lists.
