# Supplementary material for: Limited change in adolescent value-based risk choices following acute sleep restriction and caffeine
Source: Front Psychol. 2026 Aug 12;17:1879309. doi: 10.3389/fpsyg.2026.1879309 (PMC13506745; doi:10.3389/fpsyg.2026.1879309)
Supplement: Supplementary file 6 [file Supplementary_file_3.docx]

**Supplementary Figure S3 | Posterior distributions of risk decision-making outcomes**

**Figure S3.1**. Posterior distributions of regression coefficients from the hierarchical Bayesian model predicting risk-taking propensity. Density curves show posterior estimates for fixed-effect parameters, including centred and standardised age, age category, biological sex, drug condition (caffeine vs placebo), sleep condition (sleep restriction vs sleep extension), and their interaction. Black horizontal bars indicate 95% highest density intervals (HDIs), and annotated values denote posterior means. Outcomes were z-standardised prior to modelling; therefore, coefficients represent changes in standard deviation units of the outcome. Distributions overlapping zero indicate uncertainty about effect direction.

**Figure S3.2**. Posterior distributions of regression coefficients from the hierarchical Bayesian model predicting optimal choice propensity. Density curves show posterior estimates for fixed-effect parameters, including centred and standardised age, age category, biological sex, drug condition (caffeine vs placebo), sleep condition (sleep restriction vs sleep extension), and their interaction. Black horizontal bars indicate 95% highest density intervals (HDIs), and annotated values denote posterior means. Outcomes were z-standardised prior to modelling; therefore, coefficients represent changes in standard deviation units of the outcome. Distributions overlapping zero indicate uncertainty about effect direction.

**Figure S3.3**. Posterior distributions of regression coefficients from the hierarchical Bayesian model predicting irrational choice propensity. Density curves show posterior estimates for fixed-effect parameters, including centered and standardised age, age category, biological sex, drug condition (caffeine vs placebo), sleep condition (sleep restriction vs sleep extension), and their interaction. Black horizontal bars indicate 95% highest density intervals (HDIs), and annotated values denote posterior means. Outcomes were z-standardised prior to modelling; therefore, coefficients represent changes in standard deviation units of the outcome. Distributions overlapping zero indicate uncertainty about effect direction.
