# Supplementary material for: Limited change in adolescent value-based risk choices following acute sleep restriction and caffeine
Source: Front Psychol. 2026 Aug 12;17:1879309. doi: 10.3389/fpsyg.2026.1879309 (PMC13506745; doi:10.3389/fpsyg.2026.1879309)
Supplement: Supplementary file 7 [file Table_1.docx]

**Supplementary Table S1** | Participant compensation (CHF)

**Table S1.** Participant compensation (CHF)

| *Part of study* | *Participation (x = yes)* | | | | |
| --- | --- | --- | --- | --- | --- |
| Screening & Habituation | X | X | X | X | X |
| Ambulatory Phase 1 |  | X | X | X | X |
| Laboratory Phase 1 |  |  | X | X | X |
| Ambulatory Phase 2 |  |  |  | X | X |
| Laboratory Phase 2 |  |  |  |  | X |
| Partial Compensation | 20 | 35 | 45 | 55 | 65 |
| Tasks |  |  | 60 |  | 60 |
| Bonus for Completion |  |  |  |  | 160 |
| Total Compensation | 20 | 55 | 160 | 215 | 500 |

Compensation reflected time spent participating, estimated effort, and the value of the data for addressing the within-subject hypotheses. Participants earned approximately 12 CHF per task (one Monetary Incentive Delay task and four Wheel of Fortune tasks per laboratory session), totalling about 60 CHF per session. Incentivized task payments encouraged sustained engagement and optimal performance. An additional completion bonus of 160 CHF was provided, resulting in total compensation of roughly 500 CHF per participant.
