# Supplementary material for: Limited change in adolescent value-based risk choices following acute sleep restriction and caffeine
Source: Front Psychol. 2026 Aug 12;17:1879309. doi: 10.3389/fpsyg.2026.1879309 (PMC13506745; doi:10.3389/fpsyg.2026.1879309)
Supplement: Supplementary file 8 [file Table_2.docx]

**Supplementary Table S2** | Inclusion and exclusion criteria

| **Stage** |  | **Aspect** | **Assessment modality** | **Exclusion criterion** |
| --- | --- | --- | --- | --- |
| Telephone Screening |  | **Age** | MCTQ | <14years >18 years |
|  |  | **Current participation in other clinical trials** | Self-report | 'Yes' response |
|  |  | **BMI** | MCTQ | P3<BMI-PC<P97 |
|  |  | **Nicotine** | MCTQ | >0 |
|  |  | **Alcohol** | MCTQ | Use likely to have contraindications and/or effects on outcome measures |
|  |  | **History of caffeine use** | MCTQ | Habitual consumption of more than one caffeinated beverage per day (coffee, caffeinated energy drinks, or tea/iced tea), caffeine naivety, adverse withdrawal effects, or caffeine hypersensitivity; use likely to have contraindications and/or effects on outcome measures |
|  |  | **Health concerns or disorders** (heart/cardiovascular/nephrological/endocrinological/diabetic/metabolic/  chronobiologic/psychiatric/neurological [particularly epilepsy and parasomnia] conditions) | General and medical Questionnaire | Assessed case-by-case with study physician |
|  |  | **Trans meridian travel (>2 zones) <1 month prior to first session** | Self-report | 'Yes' response |
|  |  | **Shift work <3 months prior to study** | Self-report | 'Yes' response |
|  |  | **Extreme chronotype**  (mid-sleep on free days, corrected for sleep debt on workdays) | MCTQ | <3  >6 |
|  |  | **Extreme short or long sleep duration** | MCTQ | subjective sleep duration on school days <7h or >9h |
|  |  | **MRI safety** | MRI Safety Screening Questionnaire | Any 'Yes' response |
| Follow-up Screening |  | **Physical wellbeing and mental health** | KIDSCREEN-27 | T < 35 |
|  |  | **Drug use** | General and medical Questionnaire |  |
|  |  | **Medication use** | General and medical Questionnaire |  |
|  |  | **Sleep quality** | PSQI | >5 |
| In-Person Screening |  | **EEG Compatibility** | Polysomnography | Problems with EEG comfort |
|  |  | **Sleep quality** | Polysomnography | Sleep efficiency <70% |
|  |  | **Sleep disorders** | Polysomnography | Narcolepsy,  Sleep Apnoea: Index <10  Periodic limb movements: PLMI >5/h in association with high daytime sleepiness and ESS>10 |
|  |  | **BMI** | Measured height and weight | P3<BMI-PC<P97 |
|  |  | **Capacity to operate tasks and understand study language** | Interaction with experimenter | Experimenter judgement |
| Laboratory Phase of the Experimental Protocol |  | **Drug use (ALC, AMP, BZD, COC, MOR/OPI, THC, COT)** | nal von Minden  urinary drug screen | Any positive test |
|  |  | **Compliance to sleep-wake schedules** | Actigraphy record and sleep diaries | ≥1 deviation from sleep and wake-up time by >60 mins during stabilisation or >30 mins during experimental protocol |
|  |  | **Caffeine abstinence** | Sleep diaries - daily logs of caffeine intake,  and caffeine abstinence by salivary caffeine assay, with deviating participants excluded | ≥1 times caffeine intake reported in sleep diaries  ≥ 1.2 µg/mL caffeine in saliva sample on arrival at the lab quantified with a competitive salivary caffeine ELISA (Caffeine ELISA, CAFN-96; NovoLytiX GmbH, Witterswil, Switzerland) |
|  |  | **Ability to follow study instructions** | Interaction with experimenter | Experimenter judgement |
