# Supplementary material for: Limited change in adolescent value-based risk choices following acute sleep restriction and caffeine
Source: Front Psychol. 2026 Aug 12;17:1879309. doi: 10.3389/fpsyg.2026.1879309 (PMC13506745; doi:10.3389/fpsyg.2026.1879309)
Supplement: Supplementary file 9 [file Table_3.docx]

**Supplementary Table S3** | Wheel of Fortune trial types

| **Trial Type ID** | **Occurrences** | **Option A** | **Option B** | **Expected Value**  **(Option A)** | **Expected Value**  **(Option B)** | **Expected Value Relationship** |
| --- | --- | --- | --- | --- | --- | --- |
| 1 | 3 | Safe 1 | Gamble 25 | 1 | 7 | Unequal |
| 2 | 3 | Safe 3 | Gamble 8 | 3 | 7 | Unequal |
| 3 | 3 | Safe 3 | Gamble 10 | 3 | 5 | Unequal |
| 4 | 3 | Safe 3 | Gamble 15 | 3 | 7 | Unequal |
| 5 | 3 | Safe 3 | Gamble 25 | 3 | 7 | Unequal |
| 6 | 3 | Safe 5 | Gamble 8 | 5 | 7 | Unequal |
| 7 | 3 | Safe 5 | Gamble 10 | 5 | 5 | Equal |
| 8 | 3 | Safe 5 | Gamble 15 | 5 | 7 | Unequal |
| 9 | 3 | Safe 5 | Gamble 25 | 5 | 7 | Unequal |
| 10 | 3 | Safe 7 | Gamble 8 | 7 | 7 | Equal |
| 11 | 3 | Safe 7 | Gamble 10 | 7 | 5 | Unequal |
| 12 | 3 | Safe 7 | Gamble 15 | 7 | 7 | Equal |
| 13 | 3 | Safe 7 | Gamble 25 | 7 | 7 | Equal |
| 14 | 3 | Gamble 8 | Gamble 10 | 7 | 5 | Unequal |
| 15 | 3 | Gamble 8 | Gamble 15 | 7 | 7 | Equal |
| 16 | 3 | Gamble 8 | Gamble 25 | 7 | 7 | Equal |
| 17 | 3 | Gamble 10 | Gamble 15 | 5 | 7 | Unequal |
| 18 | 3 | Gamble 10 | Gamble 25 | 5 | 7 | Unequal |
| 19 | 3 | Gamble 15 | Gamble 25 | 7 | 7 | Equal |

The Wheel of Fortune comprised 57 trials derived from 19 predefined trial types. Each trial type was presented exactly three times per task, with trial order randomized. Trial types were selected without replacement from the Trial List.

Expected values for gamble options were implemented via stimulus composition rather than trial-by-trial randomisation. For each gamble magnitude, images were sampled randomly with replacement from a list containing multiple variants, each deterministically associated with either a win or a zero outcome. The expected value reported in the table reflects the proportion of winning variants within each list multiplied by the corresponding gain magnitude. Safe options always yielded the displayed amount.

The proportion of winning variants corresponded to the probability information conveyed visually by the pie-chart stimuli.
